# Neighborhood level socioeconomic disparities are associated with reduced colorectal cancer survival

**DOI:** 10.1038/s41598-025-17659-x

**Published:** 2025-09-25

**Authors:** Holli A. Loomans-Kropp, Mohamed I. Elsaid, Yesung Kweon, Anne M. Noonan, Peter P. Stanich, Jesse Plascak, Matthew F. Kalady, Chyke A. Doubeni, Electra D. Paskett

**Affiliations:** 1 Division of Cancer Prevention and Control, Department of Internal Medicine, College of Medicine, The Ohio State University, 3650 Olentangy River Road, Suite 224, Columbus, OH 43214 USA; 2 The Ohio State University Comprehensive Cancer Center, The Ohio State University, Columbus, USA; 3 Department of Biomedical Informatics, College of Medicine, The Ohio State University, Columbus, OH 43210 USA; 4 Center for Biostatistics, The Ohio State University, Columbus, OH 43210 USA; 5 Division of Medical Oncology, Department of Internal Medicine, College of Medicine, The Ohio State University, Columbus, OH 43210 USA; 6 Division of Gastroenterology, Hepatology, and Nutrition, Department of Internal Medicine, College of Medicine, The Ohio State University, Columbus, OH 43210 USA; 7 Division of Colorectal Surgery, Department of Surgery, The Ohio State University Wexner Medical Center, Columbus, OH 43210 USA; 8 Department of Family and Community Medicine, The Ohio State University Wexner Medical Center, Columbus, OH 43210 USA

**Keywords:** Mortality, Colorectal cancer, Persistent poverty, Socioeconomic status, Health outcomes, Cancer epidemiology, Gastrointestinal cancer, Risk factors

## Abstract

We evaluated the relationship between residing in persistent poverty (PP) and low socioeconomic census tracts on all-cause and colorectal cancer (CRC)-specific mortality, providing a current assessment of economic disadvantage and health outcomes. Using Surveillance, Epidemiology, and End Results Program Data (2006–2020), CRC cases were identified using ICD-10 codes and stage I-III were included in the analysis. Overlap propensity score weighting with marginal structural models estimated the risk of all-cause and CRC-specific mortality. Individuals living in PP had higher risk of all-cause mortality at 15-year follow-up, with an adjusted risk difference (ARD) and adjusted risk ratio (aRR) of 7.2 (95% CI 5.9–8.7) and 1.1 (95% CI 1.1–1.1), respectively, with similar results for CRC-specific mortality. Individuals living in low socioeconomic census tracts had higher risk of all-cause (ARD: 5.3, 95% CI 4.0–6.6; aRR: 1.1, 95% CI 1.6–1.1) and CRC-specific mortality (ARD: 2.7, 95% CI 1.7–3.7; aRR: 1.1, 95% CI 1.1–1.1) at 15-year follow-up. Thus, residing in PP or low socioeconomic census tract may impact health outcomes.

**Supplementary Information:**

The online version contains supplementary material available at 10.1038/s41598-025-17659-x.

## Introduction

In 2024, it was projected that over 150,000 people will be diagnosed with colorectal cancer (CRC) in the United States (U.S.), and approximately 50,000 would die from this disease^1^. CRC outcomes vary according to measures of social disadvantage, including place of residence. Similarly, geographic disparities of CRC outcomes are well-documented, with evidence suggesting that residential socioeconomic deprivation around the time of diagnosis is associated with worse survival outcomes^2,3^. Recent studies have also sought to understand the multi-factorial nature of socioeconomic deprivation, showing that Persistent Poverty (PP), defined as a census tract where ≥ 20% or more of the population has lived below the poverty level for approximately 30 years, at the county level is associated with adverse cancer outcomes^4,5^. Furthermore, PP may be a more accurate measure of cumulative disadvantage than current poverty status or individual socioeconomic status (SES; defined as a composite measure of income, poverty, education, employment, occupation, home ownership, etc.), as PP represents chronic poverty exposure and associated risk factors^6^. Despite a decrease in the number of counties in the U.S. classified as PP, roughly 28 million people continue to reside in PP^7,8^.

PP has been associated with chronic diseases, including cancer, and adverse health outcomes, including overall mortality and cancer-specific mortality^4,9–12^. In an analysis of 2007–2011 SEER data, residing in PP areas was associated with a higher risk of overall cancer and CRC-specific mortality risk than in non-PP areas, while a separate study observed that age-adjusted all-cancer and site-specific cancer mortality rates were substantially higher, as well as lower survival, for those living in poverty, with similar results seen at the state-level^4,5,13–15^. CRC is a prime example of where improvements in impoverishment, SES or reductions in disparities, such as better access to screening for earlier diagnosis and treatments can directly impact patient outcomes^16^. Many studies have established the connection between economic disadvantage and adverse CRC outcomes, often relying on area-level metrics or individual measures of socioeconomic deprivation^4,5,17,18^. However, PP and SES are intertwined, despite PP being static and SES being dynamic, and PP being causally associated with SES. Utilizing the area-level or individual metrics may oversimplify the complex relationship between PP and SES and hinder the application of findings to targeted interventions. Thus, we employed an analytical technique, using population-based data, to overcome these limitations to aid in developing strategies for targeted intervention.

In our statistical analysis, we utilized a comprehensive causal inference approach to more accurately estimate the relationships between PP and socioeconomic census tract in our assessments of all-cause and CRC-specific mortality. In the current study, we utilized the most recent data release from SEER Research Plus Specialized Data (1995–2020), with data from 17 registries and census tract attributes, to investigate these associations, evaluating the relationships PP and census tract-level SES on overall and CRC-specific mortality, providing an up-to-date assessment of economic disadvantage and adverse health outcomes. This study investigates the directionality and interplay of PP and SES in influencing overall and cancer outcomes.

## Methods

This population-based retrospective cohort study used SEER data, a national comprehensive registry of annual cancer incidence and mortality covering nearly half of the United States population^19^. For this analysis, we used SEER Research Plus Specialized Data, which includes data from 17 registries and census tract attributes^20^. The specialized data has census tract-based measures, such as rural–urban status, area-level PP, and SES, in addition to characteristics, such as patient demographics, tumor features, initial treatment information, and vital status, found in the standard SEER data. This analysis used de-identified, publicly available data and was considered exempt from institutional review board review. We adhered to STROBE guidelines for observational studies^21^.

### Study population

Newly diagnosed individuals aged 18 and older with CRC from 2006 to 2020 were identified using the International Classification of Disease 10 (ICD-10) codes C18.0-C18.9. C19.9, and C20.9 with histology codes. Proximal cancers were those diagnosed in the cecum (C18.0), ascending colon (C18.2), hepatic flexure (C18.3), and transverse colon (C18.4) and distal cancers were those diagnosed in the splenic flexure (C18.5), descending colon (C18.6), and sigmoid colon (C18.7). Codes for overlapping lesions or those with unspecified origin were excluded from subsite analysis. Exclusion criteria for the analysis was (1) American Joint Committee on Cancer (AJCC) stage IV CRC, (2) SEER Summary stage of distant or metastatic disease, (3) unknown surgery status, (4) cancer diagnosis reported on autopsy or death, (5) unknown cause of death, and (6) inaccurate geocodes. We additionally excluded individuals with unknown urban-area categorization status for analysis in the PP analysis (PP cohort) and unknown socioeconomic census tract status for the SES analysis (SES cohort).

### Definitions of exposures

We included two main exposures of interest in this study: (1) residing in a PP census tract (PP cohort) and (2) residing in a low socioeconomic census tract at diagnosis (SES cohort). PP is defined as a census tract where 20% or more of the population has lived below the poverty level for a span of approximately 30 years^22^. PP status was determined from the 1990 and 2000 decennial Censuses and the 2007–2011 and 2015–2019 American Community Survey (ACS) 5-year estimates, whereas the Yost SES quintile was assigned using the ACS 5-year file contemporaneous with each patient’s diagnosis year; thus, persistent poverty captures decades-long deprivation that temporally precedes, and can causally shape, the tract’s SES at diagnosis. This variable categorizes census tracts as residing or not residing in PP census tract. Neighborhood SES was supplied by SEER as the Yost index quintile and were calculated using composite SES scores using a factor analysis from median household income, median house value, median rent, percent below 150% of the poverty line, education index (median education, median years of school, percent who completed high school or college)^23^, percent working class, and percent unemployed^6^. Cancer cases were linked to the SES quintiles by diagnosis year and corresponding ACS five-year estimate. The index was categorized into quintiles, with the lowest SES group as first quintile.

### Assessment of outcomes

The primary outcome of interest was time to CRC-specific mortality and the secondary outcome was time to all-cause mortality, derived from SEER’s standardized survival data and includes follow-up time and cause of death, if applicable. Follow-up began at the date of entry into the cohort and continued until the recorded date of death, date of last follow-up, or the end of the study (12/31/2020), whichever came first. In the CRC-specific analyses, patients were censored on the date of the non-CRC event, if the cause of death was not due to CRC.

### Variables of interest

We extracted sociodemographic, clinical and treatment variables from SEER, specifically age, sex, race, ethnicity, marital status, year of diagnosis, and census urban-area categorization. Clinical and treatment variables included CRC histology type, pathologic grade, SEER summary and AJCC stage information (including tumor/node/metastasis (TNM) status), primary tumor size, number of tumors, surgery status, receipt of chemotherapy or radiation, and time from diagnosis to the start of treatment.

### Approach to confounding

We were interested in examining the causal relationships between exposures (PP, census tract-level SES), confounders, and outcomes (all-cause, CRC-specific mortality) and used directed acyclic graphs to map these relationships (Supplementary Figure S1). In the SES analysis, the SES census tract was considered a mediator of the causal relationship between PP and mortality. As PP is a fixed area-level exposure, it precedes and is causally associated with SES census tract status. As we were interested in defining total effects, adjustments were not made for SES census tract status in the evaluation of PP on survival. By total effect, we mean the combined direct impact of persistent poverty on mortality and its indirect impact mediated through contemporaneous tract-level SES and other downstream factors; therefore, SES was not conditioned on when estimating PP’s total effect. In the assessment of SES on survival, PP was considered a confounder between SES census tract status and mortality, as PP is associated with the exposure (SES census tract), is a risk factor of the outcome, and is not in the causal pathway between exposure and outcome.

### Statistical analysis

We used overlap propensity score weighting (OPSW) to account for the impact of confounding resulting from the imbalances in baseline sociodemographic, clinical, and treatment variables between exposure groups^24^. OPSW is a method that imitates important features of randomized clinical trials, including covariate balance and statistical precision, compared to other weighting methods, like Inverse Probability Treatment Weighting (IPTW)^24,25^. OPSW allows for a balance of means and proportion of each variable included in the logistic regression models used to estimate the propensity score, which we used in the analysis of PP and SES in two separate logistic regression models, with mortality as the outcome and variables, respectively. We used the forward effect selection method to improve model fit. Overlap weights were estimated separately for the PP and SES cohorts using estimated propensity scores from each cohort.

Within each cohort, overall and stratified characteristics were summarized with descriptive statistics, including means with corresponding standard deviations (SD) for continuous variables and frequencies and proportions for categorical variables. Differences in exposures were compared using Student’s *t*-tests or Wilcoxon Rank Sum tests for continuous variables and chi-square or Fisher’s exact tests for categorical variables, whichever was most appropriate. Overlap-weighted standardized mean differences, which assess covariate balance, were used to compare sociodemographic, clinical, and treatment variables, by exposure status. A standardized difference of less than 0.1 indicated negligible differences between patient characteristics and exposure status^26^.

We fitted marginal structural models using parametric pooled logistic regression—which allows for the controlling of variables that change over time—including an indicator for the exposure group, a flexible time-varying intercept, and interaction terms between the exposure group (PP, SES) and time^27^. By employing marginal structural models with parametric pooled logistic regression, we could flexibly account for time-varying hazards and interactions between exposure(s) and time, leading to a more accurate estimation of the causal effects of PP and census tract SES status on CRC survival. All marginal structural models were weighted using OPSW. The average 1-, 5-, 10-, and 15-year absolute all-cause and CRC-specific mortality risks for each exposure group were estimated using the predicted values from the weighted marginal structural models. The resulting risk differences (RDs) and risk ratios (RRs) were calculated. We used non-parametric bootstrapping with 1000 replications to estimate 95% confidence intervals (CIs) for RDs and RRs^28^. Cox proportional hazard models assume a constant hazard ratio over time and often report a single, weighted average hazard ratio, which can obscure time-varying effects and complicate causal interpretation due to potential selection bias from conditioning on survival^29,30^.

Secondary analyses were conducted to enhance the generalizability and comparability of our findings. All-cause and CRC-specific mortality differences were assessed using Kaplan–Meier analysis. Differences in crude absolute incidence rate (expressed as person time) for all outcomes were calculated for exposed and unexposed groups. Crude and OPSW Cox proportional hazards regression models were fitted to assess the relationships between PP and SES census tract with all-cause and CRC-specific mortality. Sensitivity analyses were completed to extend the study cohorts (PP, SES) to include participants with AJCC stage IV CRC and distant or metastatic disease, repeating the OPSW proportional hazards regression analyses to the expanded cohorts. Two-sided tests were considered statistically significant at a significance level of 0.05. All analyses were completed using SAS version 9.4 and R 4.2.0.

## Results

### PP and SES census tract level cohort characteristics

**Fig. 1:**
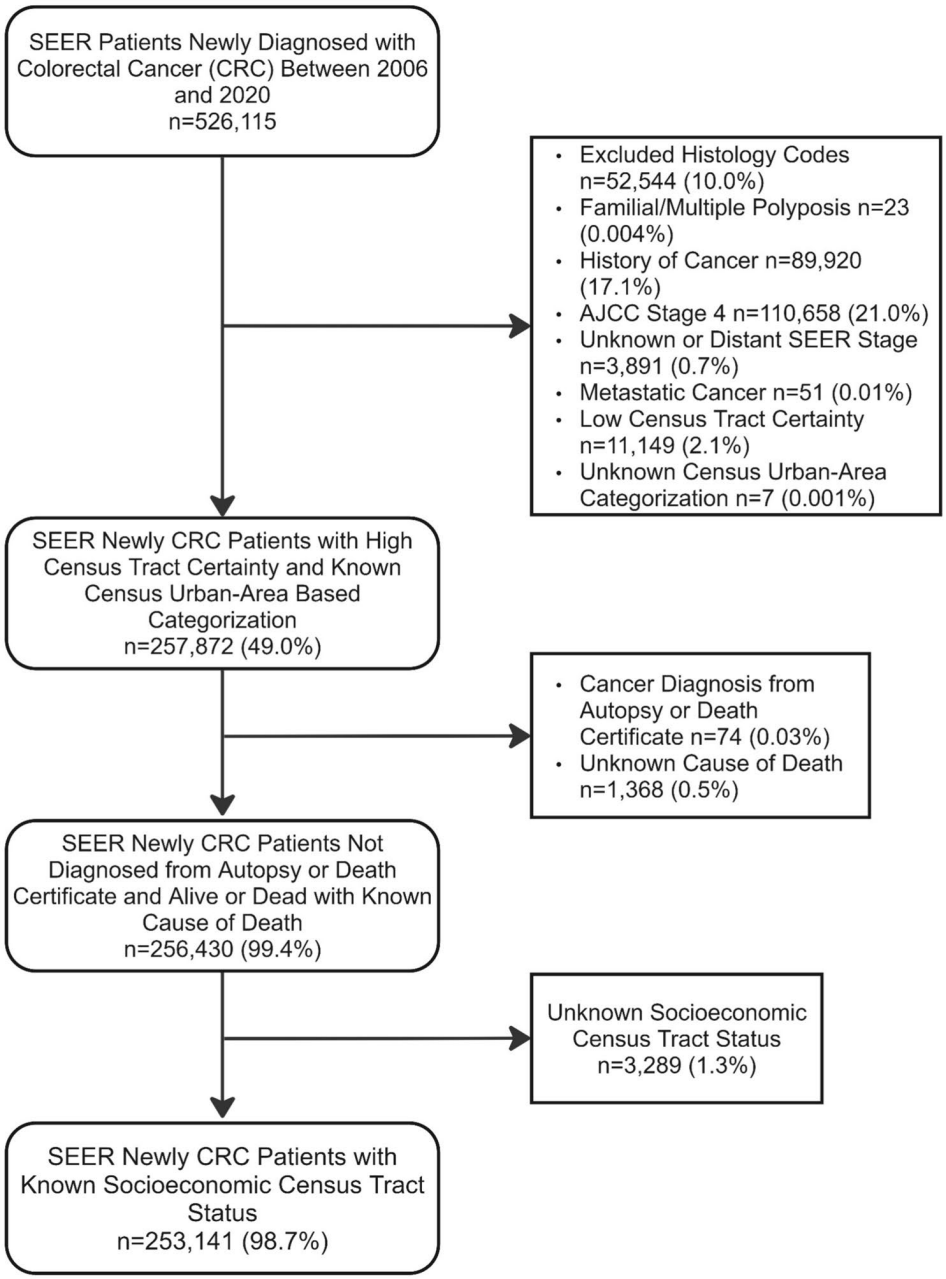
Flowchart outlining eligible participants from the Surveillance, Epidemiology, and End Results (SEER) Program Data. From the initial cohort of newly diagnosed CRC cases between 2006 and 2020 (n = 526,115), 257,872 had census tract and urban-area information. After excluding those with cancer diagnosis at death or unknown cause of death, and unknown socioeconomic status, 253,141 participants were eligible for our study.

Between 2006 and 2020, 526,115 diagnoses of CRC were reported in the SEER database (Fig. 1). After excluding for histology (N = 52,544) and heritable causes of CRC (N = 23), we had a study population of 473,548. We further restricted our study to individuals with prior a cancer diagnosis (N = 89,920) and late-stage cancer (N = 114,600) and excluded those without a noted census tract (N = 11,149) or area characterization (N = 7), cancer diagnosed at the time of death (N = 74) or unknown cause of death (N = 1,368), and unknown SES status (N = 3,289). Our eligible population consisted of 253,141 individuals.

The majority of the eligible cohort did not reside in census tracts with PP (91.5%) and other sociodemographic, clinical, and treatment characteristics were significantly different between those residing in PP and those not residing in PP (Table 1). Compared to those not living in PP, those residing in persistent poverty were more likely to be non-Hispanic Black (32.5% v. 8.4%; p < 0.0001) and less likely to be married or partnered (41.9% v. 55.6%; p < 0.0001) and living in all urban or mostly urban census tracts (84.6% v. 86.5%; p < 0.0001). Those living in PP with CRC were less likely to have adenocarcinoma histology (92.1% v. 92.4%; p < 0.0001), diagnosed in the proximal colon (41.5%) and more likely to present with regional stage (53.5% v. 52.7%; p = 0.03), and tumor stage T3 (48.3% v. 47.1%; p < 0.0001) (Table 1). Similar demographics were observed in the socioeconomic census tract-selected population (Supplementary Table S1). We then used overlap weighting of variables with a standardized difference greater than 0.1 to balance the PP cohort for additional analyses (Table 1). The same characteristics were used to weight and select for the socioeconomic census tract analyses (Supplementary Table S1).

**Table 1:** Sociodemographic, Clinical, and Treatment Characteristics of Patients with Colorectal Cancer by Residence in Persistent Persistently Impoverished Census Tracts Status, The Surveillance, Epidemiology, and End Results (SEER) 2006–2020 (n = 256,430).

Patient Characteristics	Unweighted			Weighted*		
	Not Residing in Persistently Impoverished Census Tract	Residing in Persistently Impoverished Census Tract	Standardized Difference^†^	Not Residing in Persistently Impoverished Census Tract	Residing in Persistently Impoverished Census Tract	Standardized Difference^§^
	n (%)	n (%)		%^‡^	%^‡^	
No. of Patients	234,522 (91.5%)	21,908 (8.5%)		50.0%	50.0%	
Age						
Mean (SD)	66.2 (13.74)	64.9 (13.10)	0.090	65.2 (3.8)	65.1 (12.2)	0.010
Median (IQR)	66.0 (56.0, 77.0)	65.0 (56.0, 75.0)		65.0 (56.0, 75.0)	65.0 (56.0, 75.0)	
Age Groups, n (%)						0^||^
20–50 years	31,879 (13.6%)	2981 (13.6%)	0.000	13.7%	13.7%	
51–60 years	49,383 (21.1%)	5189 (23.7%)	0.063	23.3%	23.3%	
61–70 years	60,072 (25.6%)	6133 (28.0%)	0.054	27.7%	27.7%	
70 + years	93,188 (39.7%)	7605 (34.7%)	0.104	35.4%	35.4%	
Sex, n (%)						0^||^
Male	121,912 (52.0%)	11,470 (52.4%)	0.010	52.4%	52.4%	
Female	112,610 (48.0%)	10,438 (47.6%)	0.010	47.6%	47.6%	
Race-Ethnicity, n (%)						0^||^
Non-Hispanic White	163,316 (69.6%)	8971 (40.9%)	0.603	45.1%	45.1%	
Non-Hispanic Black	19,813 (8.4%)	7123 (32.5%)	0.625	27.6%	27.6%	
Non-Hispanic American Indian/Alaska Native	997 (0.4%)	121 (0.6%)	0.018	0.6%	0.6%	
Non-Hispanic Asian or Pacific Islander	22,469 (9.6%)	1322 (6.0%)	0.132	6.7%	6.7%	
Hispanic	26,811 (11.4%)	4325 (19.7%)	0.231	19.8%	19.8%	
Non-Hispanic Unknown Race	1116 (0.5%)	46 (0.2%)	0.045	0.2%	0.2%	
Marital Status at Diagnosis, n (%)						0^||^
Married or Partner	130,436 (55.6%)	9175 (41.9%)	0.277	43.9%	43.9%	
Separated or Divorced	23,060 (9.8%)	2795 (12.8%)	0.093	12.4%	12.4%	
Single Never Married	33,931 (14.5%)	5360 (24.5%)	0.255	22.8%	22.8%	
Widowed	35,485 (15.1%)	3371 (15.4%)	0.007	15.4%	15.4%	
Unknown	11,610 (5.0%)	1207 (5.5%)	0.025	5.5%	5.5%	
Year of Diagnosis, n (%)						0^||^
2006	16,706 (7.1%)	1458 (6.7%)	0.018	6.7%	6.7%	
2007	16,806 (7.2%)	1489 (6.8%)	0.014	6.8%	6.8%	
2008	16,693 (7.1%)	1507 (6.9%)	0.009	6.9%	6.9%	
2009	16,424 (7.0%)	1533 (7.0%)	0.000	7.0%	7.0%	
2010	15,818 (6.7%)	1508 (6.9%)	0.005	6.8%	6.8%	
2011	15,677 (6.7%)	1504 (6.9%)	0.007	6.8%	6.8%	
2012	16,014 (6.8%)	1480 (6.8%)	0.003	6.8%	6.8%	
2013	15,356 (6.5%)	1429 (6.5%)	0.001	6.5%	6.5%	
2014	16,043 (6.8%)	1589 (7.3%)	0.016	7.2%	7.2%	
2015	15,783 (6.7%)	1541 (7.0%)	0.012	7.1%	7.1%	
2016	15,469 (6.6%)	1468 (6.7%)	0.004	6.7%	6.7%	
2017	15,154 (6.5%)	1369 (6.2%)	0.009	6.3%	6.3%	
2018	14,583 (6.2%)	1463 (6.7%)	0.019	6.6%	6.6%	
2019	15,153 (6.5%)	1398 (6.4%)	0.003	6.4%	6.4%	
2020	12,843 (5.5%)	1172 (5.3%)	0.006	5.4%	5.4%	
Census Urban-Area Categorization, n(%)						0^||^
All Urban	154,034 (65.7%)	14,231 (65.0%)	0.015	64.6%	64.6%	
Mostly Urban	48,754 (20.8%)	4299 (19.6%)	0.029	19.8%	19.8%	
Mostly Rural	16,524 (7.0%)	1322 (6.0%)	0.041	6.3%	6.3%	
All Rural	15,210 (6.5%)	2056 (9.4%)	0.107	9.3%	9.3%	
Clinical and Treatment Characteristics						
Colorectal Cancer Histological Type, n (%)						0^||^
Adenocarcinoma	216,747 (92.4%)	20,219 (92.1%)	0.005	92.3%	92.3%	
Mucinous	16,047 (6.9%)	1566 (7.2%)	0.012	7.1%	7.1%	
Other	1728 (0.6%)	123 (0.6%)	0.022	0.6%	0.6%	
SEER Summary Stage, n (%)						0^||^
Localized	110,902 (47.3%)	10,191 (46.5%)	0.020	46.5%	46.5%	
Regional	123,620 (52.7%)	11,717 (53.5%)	0.020	53.5%	53.5%	
AJCC Stage, n (%)						0^||^
I	72,534 (30.9%)	6308 (28.8%)	0.047	29.0%	29.0%	
II	76,555 (32.6%)	7493 (34.2%)	0.033	34.0%	34.0%	
III	85,433 (36.4%)	8107 (37.0%)	0.012	37.0%	37.0%	
Pathologic Grade, n (%)						
Grade 1	20,118 (8.6%)	1849 (8.4%)	0.005	8.6%	8.4%	0.009
Grade 2	152,110 (64.9%)	14,279 (65.2%)	0.007	65.1%	65.1%	0.002
Grade 3	28,649 (12.2%)	2421 (11.1%)	0.036	11.6%	11.1%	0.013
Grade 4	4060 (1.7%)	349 (1.6%)	0.011	1.7%	1.6%	0.002
Unknown	29,585 (12.6%)	3010 (13.7%)	0.033	13.1%	13.7%	0.018
TNM-T, n (%)						0^||^
T0/T1	42,467 (18.1%)	3700 (16.9%)	0.032	17.0%	17.0%	
T2	33,250 (14.2%)	2888 (13.2%)	0.029	13.3%	13.3%	
T3	110,435 (47.1%)	10,577 (48.3%)	0.024	48.1%	48.1%	
T4	5853 (2.5%)	530 (2.4%)	0.005	2.4%	2.4%	
Tx	42,517 (18.1%)	4213 (19.2%)	0.028	19.2%	19.2%	
TNM-N, n (%)						0^||^
N0	134,300 (57.3%)	12,472 (56.9%)	0.007	56.9%	56.9%	
N1	21,704 (9.3%)	1918 (8.8%)	0.017	8.8%	8.8%	
Nx	78,518 (33.5%)	7518 (34.3%)	0.018	34.3%	34.3%	
Surgery Status, n (%)						
None	10,000 (4.3%)	1196 (5.5%)	0.056	4.6%	5.4%	0.035
Local tumor surgery	57,439 (24.5%)	4902 (22.4%)	0.050	23.3%	22.6%	0.016
Colectomy or proctectomy	166,954 (71.2%)	15,787 (72.1%)	0.019	72.0%	71.8%	0.003
Unknown	129 (0.1%)	23 (0.1%)	0.018	0.1%	0.1%	0.014
Radiotherapy, n (%)						
No/Unknown¶	196,403 (83.7%)	18,340 (83.7%)	0.001	83.8%	83.6%	0.006
Yes	37,171 (15.8%)	3461 (15.8%)	0.001	15.8%	15.9%	0.002
Refusal	948 (0.4%)	107 (0.5%)	0.013	0.4%	0.5%	0.018
Chemotherapy, n (%)						
No/Unknown	147,134 (62.7%)	13,738 (62.7%)	0.001	61.8%	62.8%	0.020
Yes	87,388 (37.3%)	8170 (37.3%)	0.001	38.2%	37.2%	0.020
Time from Diagnosis to Treatment (Months), n(%)						
Mean (SD)	0.7 (1.03)	0.7 (1.19)	0.030	0.7 (0.3)	0.7 (1.1)	0.010
Median (IQR)	0.0 (0.0, 1.0)	0.0 (0.0, 1.0)		0 (0, 1)	0 (0, 1)	
Time from Diagnosis to Treatment (Months), n(%)						
1 month or less	197,456 (84.2%)	17,863 (81.5%)	0.071	82.9%	81.6%	0.032
2 months	23,042 (9.8%)	2194 (10.0%)	0.006	10.2%	10.0%	0.008
3 months or more	8912 (3.8%)	1164 (5.3%)	0.073	4.5%	5.2%	0.033
Unknown	5112 (2.2%)	687 (3.1%)	0.059	2.4%	3.1%	0.047
Tumor Size (mm)						
Mean (SD)	45.3 (33.68)	47.7 (34.22)	0.070	47.1 (10)	47.5 (31.6)	0.010
Median (IQR)	40.0 (27.0, 60.0)	45.0 (30.0, 60.0)		43 (30, 60)	44 (30, 60)	
Tumor Size Group, n (%)						0^||^
< 50	126,606 (54.0%)	10,994 (50.2%)	0.076	50.7%	50.7%	
≥ 50	80,163 (34.2%)	8208 (37.5%)	0.069	37.0%	37.0%	
Unknown	27,753 (11.8%)	2706 (12.4%)	0.016	12.3%	12.3%	
Tumor Number of in situ/malignant Tumors						
Mean (SD)	1.2 (0.44)	1.2 (0.42)	0.030	1.2 (0.1)	1.2 (0.4)	0.010
Median (IQR)	1.0 (1.0, 1.0)	1.0 (1.0, 1.0)		1 (1, 1)	1 (1, 1)	
Total Number of in situ/malignant Tumors						0^||^
1	201,403 (85.9%)	18,997 (86.7%)	0.020	86.7%	86.7%	
2 +	33,119 (14.1%)	2911 (13.3%)	0.020	13.3%	13.3%	
Tumor Location, n (%)						0^||^
Proximal colon	98,302 (41.9%)	9096 (41.5%)	0.008	41.5%	41.5%	
Distal colon	65,032 (27.7%)	6405 (29.2%)	0.033	29.0%	29.0%	
Other	71,188 (30.4%)	6407 (29.2%)	0.024	29.5%	29.5%	

*Using overlap weighting. The weighting aims to construct a pseudo-sample in which persistent poverty status is independent of the baseline demographics and cancer characteristics influencing the likelihood of residing in a persistent poverty census tract.

^†^Absolute difference in means or proportions divided by pooled standard deviation. The imbalance between the persistent poverty census tract and not persistent poverty census tract groups is defined as an absolute value greater than 0.10; smaller values indicate better balance.

^‡^Overlap weighted proportions.

^§^Overlap-weighted standardized differences. All patient baseline demographics and cancer characteristics were used to estimate the weights.

^||^Overlapping weighting resulted in an exact balance for this variable.

^¶^Including recommended and unknown if administered.

*SD* standard deviation.

### Association of residing in PP on all-cause and CRC-specific mortality

**Fig. 2:**
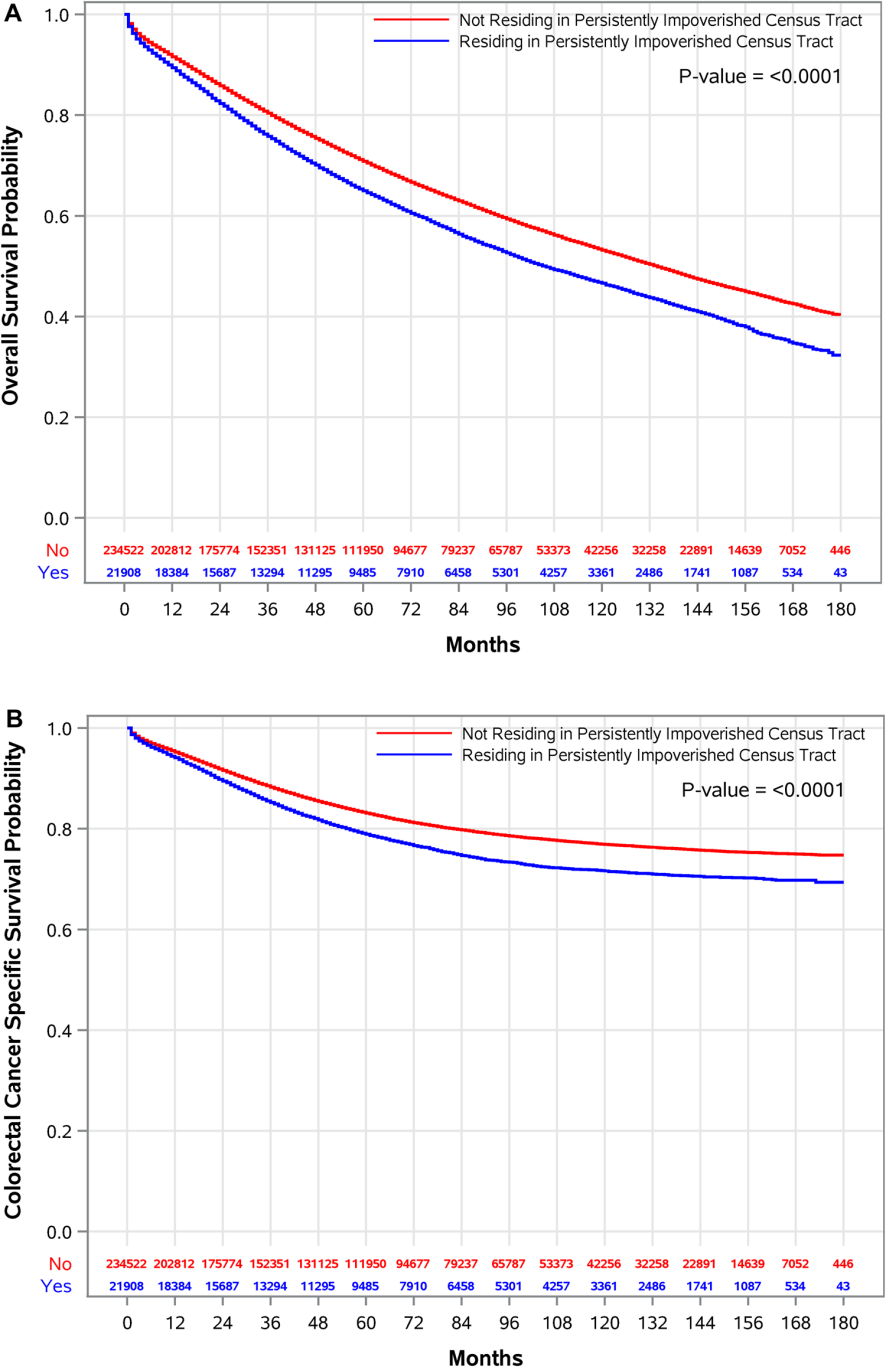
Survival probabilities of study participants residing in PP, compared to those not residing in PP. Using OPSW-weighted marginal structural models, we estimated average 1-, 5-, 10-, and 15-year absolute (**A**) all-cause and (**B**) CRC-specific mortality risks for those residing in persistently impoverished census tracts (PP) compared to those not residing in persistently impoverished census tracts.

We observed significantly increased risk of all-cause mortality was observed at 1- (ARD, 1.9%; 95%CI, 1.6–2.3), 5- (ARD, 4.5%; 95%CI, 3.8–5.2), 10- (ARD, 7.2%; 95%CI, 4.6–6.3), and 15-years of follow-up (ARD, 7.2; 95%CI, 5.9–8.7) (Table 2) for individuals living in PP. After 15 years of follow-up, all-cause mortality risk was 12% (aRR, 1.12; 95%CI, 1.10–1.14) higher for those residing in PP, compared to those not residing in PP. Similar results were observed for CRC-specific mortality, where an ARD of 2.9% observed in 15 years of follow-up (95%CI, 1.9–3.9) for individuals living in PP. In the first year of follow-up, adjusted risk for CRC-specific mortality was 16% higher for those residing in PP (95%CI, 1.11–1.23), compared to non-PP, and 10% higher after 15 years of follow-up (aRR, 1.10; 95%CI, 1.07–1.14). Cumulative risk of overall and CRC mortality is shown in Supplementary Figure S2A,B. Residing in PP was associated with increased hazard of all-cause (aHR, 1.18; 95%CI, 1.16–1.21) and CRC-specific (aHR, 1.15; 95%CI, 1.11–1.18) mortality (Supplementary Table S2). Kaplan–Meier survival curves showed significantly lower survival for overall (p < 0.0001) and CRC-specific (p < 0.0001) mortality for those living in PP, compared to those not in PP census tracts (Fig. 2).

**Table 2:** Estimated 15-Year Overlapping Weight Adjusted Risks* of All-Cause and Colorectal Cancer Specific Mortalities Comparing Patients Residing vs. Not Residing in a Persistently Impoverished Census Tract, Patients with Colorectal Cancer, The Surveillance, Epidemiology, and End Results (SEER), 2006–2020 (n = 256,430).

Follow-Up (Years)	Adjusted Cumulative Risk (%) (95%CI)		Adjusted Risk Difference^†^ (%) (95%CI)	Adjusted Risk Ratio^‡^ (95%CI)
	Persistent Poverty Census Tract	Not Persistent Poverty Census Tract		
	All-Cause Mortality			
1	11.1 (10.7 to 11.4)	9.1 (9.0 to 9.3)	1.9 (1.6 to 2.3)	1.21 (1.17 to 1.25)
5	35.6 (35.0 to 36.3)	31.1 (30.8 to 31.4)	4.5 (3.8 to 5.2)	1.14 (1.12 to 1.17)
10	53.2 (52.5 to 54.1)	47.8 (47.4 to 48.2)	5.4 (4.6 to 6.3)	1.11 (1.10 to 1.13)
15	68.5 (67.2 to 70.0)	61.3 (60.7 to 61.9)	7.2 (5.9 to 8.7)	1.12 (1.10 to 1.14)
	Colorectal Cancer Specific Mortality			
1	6.5 (6.2 to 6.8)	5.5 (5.4 to 5.7)	0.9 (0.6 to 1.2)	1.16 (1.11 to 1.23)
5	21.1 (20.6 to 21.8)	18.6 (18.4 to 18.9)	2.5 (1.9 to 3.1)	1.13 (1.10 to 1.17)
10	28.3 (27.5 to 29.1)	25.4 (25.1 to 25.7)	2.9 (2.1 to 3.7)	1.11 (1.08 to 1.15)
15	30.3 (29.3 to 31.4)	27.4 (27.0 to 27.8)	2.9 (1.9 to 3.9)	1.10 (1.07 to 1.14)

*Adjusted using overlapping weights estimated using sex, age, race-ethnicity, marital status at diagnosis, year of diagnosis, census urban-area categorization, colorectal cancer histological type, seer summary stage, AJCC staging, TNM-N, TNM-T, tumor location, tumor size, and total number of in situ/malignant tumors.

^†^Difference between residing and not residing in a persistently impoverished census tract.

^‡^Comparing those residing vs. not residing in a persistently impoverished census tract.

*CI* confidence interval.

We completed sensitivity analyses, including CRC cases that were stage IV or had an unknown stage, distant disease, or metastases (M1), and the sample size increased to 364,510 for the PP cohort. For those living in PP, the all-cause crude and overlap weighting (OW)-adjusted HRs were 1.25 (95%CI, 1.23–1.27) and 1.13 (95%CI, 1.11–1.15), respectively, after 15 years of follow-up, compared to those not living in PP, and the crude and OW-adjusted HR for CRC-specific mortality was 1.26 (95%CI, 1.23–1.28) and 1.09 (95%CI, 1.06–1.11) (Supplementary Table S3). These estimates are similar to those observed in the initial analyses.

### Association of SES census tract-level on all-cause and CRC-specific mortality

**Fig. 3:**
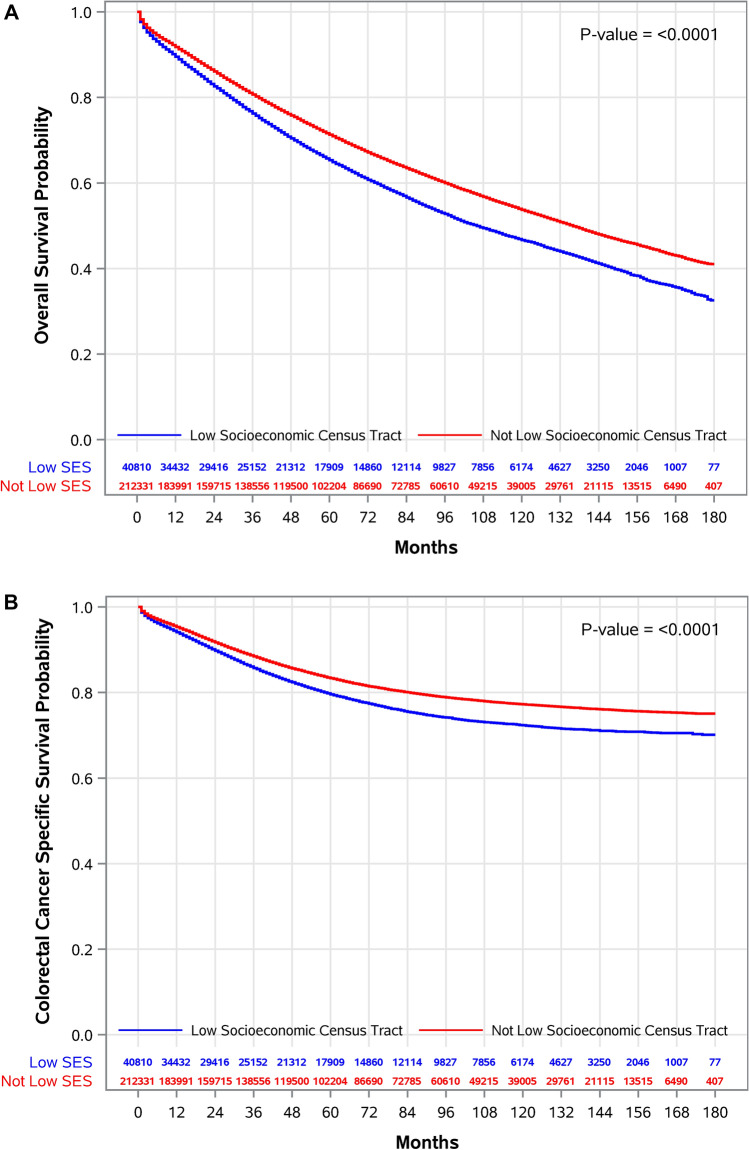
Survival probabilities of study participants living in low SES census tracts, compared to those not living in low SES census tracts. Using OPSW-weighted marginal structural models, we estimated average 1-, 5-, 10-, and 15-year absolute (**A**) all-cause and (**B**) CRC-specific mortality risks for those residing in low SES census tracts compared to those not residing in low SES census tracts.

When we examined cumulative risk of overall and CRC-specific mortality, comparing those residing in low socioeconomic census tracts to those not in low socioeconomic census tracts, significantly higher risk of all-cause mortality was observed at 1- (ARD, 1.5%; 95%CI, 1.1–1.8), 5- (ARD, 4.1%; 95%CI, 3.5–4.6), 10- (ARD, 5.1%; 95%CI, 4.2–5.9), and 15-years of follow-up (ARD, 5.3; 95%CI, 4.0–6.6) (Table 3**, **Supplementary Figure S2C,D). All-cause mortality risk after 15 years of follow-up was 9% (aRR, 1.09; 95%CI, 1.06–1.11) higher in low socioeconomic census tracts, compared to those not in low socioeconomic census tracts. Likewise, for CRC-specific mortality, ARD after 15 years of follow-up was 2.7% (95%CI, 1.7–3.7) for individuals living in low socioeconomic census tracts. Relative risk of CRC-specific mortality was 13% (aRR, 1.13; 95% CI, 1.08–1.19) and 10% (aRR, 1.10; 95%CI, 1.06–1.14) higher among those in low socioeconomic census tracts in the first year of follow-up and after 15 years of follow-up, respectively (Table 3). Residing in low socioeconomic census tract was associated with increased hazard of all-cause (aHR, 1.16; 95%CI, 1.14–1.19) and CRC-specific (aHR, 1.12; 95%CI, 1.09–1.16) mortality (Supplementary Table S2). Similar to PP, significantly lower survival for overall (p < 0.0001) and CRC-specific (p < 0.0001) mortality was observed for those residing in low SES census tracts, compared to those not residing in low SES census tracts (Figs. 2,3).

**Table 3:** Estimated 15-Year Overlapping Weight Adjusted Risks* of All-Cause and Colorectal Cancer Specific Mortalities Comparing Patients Residing vs. Not Residing in Low Socioeconomic Census Tract, Patients with Colorectal Cancer, The Surveillance, Epidemiology, and End Results (SEER) 2006–2020 (n = 256,430).

Follow-Up (Years)	Adjusted Cumulative Risk (%) (95%CI)		Adjusted Risk Difference^†^ (%) (95%CI)	Adjusted Risk Ratio^‡^ (95%CI)
	Residing in Low Socioeconomic Census Tract	Not Residing in Low Socioeconomic Census Tract		
	All-Cause Mortality			
1	10.6 (10.3 to 10.9)	9.1 (8.9 to 9.3)	1.5 (1.1 to 1.8)	1.16 (1.12 to 1.20)
5	35.0 (34.4 to 35.6)	30.9 (30.6 to 31.3)	4.1 (3.5 to 4.6)	1.13 (1.11 to 1.15)
10	52.8 (52.1 to 53.5)	47.7 (47.3 to 48.1)	5.1 (4.2 to 5.9)	1.11 (1.09 to 1.12)
15	67.2 (66.1 to 68.5)	62.0 (61.4 to 62.6)	5.3 (4.0 to 6.6)	1.09 (1.06 to 1.11)
	Colorectal Cancer Specific Mortality			
1	6.3 (6.0 to 6.6)	5.6 (5.4 to 5.7)	0.7 (0.5 to 1.0)	1.13 (1.08 to 1.19)
5	20.0 (19.5 to 20.5)	18.1 (17.8 to 18.4)	1.9 (1.4 to 2.4)	1.11 (1.07 to 1.14)
10	26.9 (26.2 to 27.6)	24.5 (24.2 to 24.9)	2.4 (1.7 to 3.1)	1.10 (1.07 to 1.13)
15	29.4 (28.6 to 30.4)	26.8 (26.4 to 27.3)	2.7 (1.7 to 3.7)	1.10 (1.06 to 1.14)

*Adjusted using overlapping weights estimated using sex, age, race-ethnicity, marital status at diagnosis, year of diagnosis, census urban-area categorization, colorectal cancer histological type, seer summary stage, AJCC staging, TNM-N, TNM-T, tumor location, tumor size, total number of in situ/malignant tumors, and residing persistently impoverished census tract.

^†^Difference between residing and not residing in a persistently impoverished census tract.

^‡^Comparing those residing vs. not residing in a persistently impoverished census tract.

*CI* confidence interval.

Like the PP cohort, we completed sensitivity analyses for the SES cohort, with the sample size increasing to 364,510 with the inclusion of CRC stage IV or unknown stage, distant disease, or metastasis. The crude and OW-adjusted HRs for all-cause mortality among those living in low SES census tracts were 1.23 (95%CI, 1.22–1.25) and 1.12 (95%CI, 1.10–1.14), respectively, after 15 years of follow-up, compared to those not in low SES census tracts, and the crude and OW-adjusted HR for CRC-specific mortality was 1.22 (95%CI, 1.20–1.24) and 1.07 (95%CI, 1.05–1.10), respectively, after 15 years of follow-up, similar to our previous analyses (Supplementary Table S3).

## Discussion

We showed that living in PP was associated with higher all-cause and CRC-specific mortality 15 years post-diagnosis. Similarly, compared to those living in high SES tracts, residing in low SES census tracts was associated with lower all-cause and CRC-specific mortality. Our findings in this study are consistent with the literature, suggesting the inter-relatedness of poverty and health outcomes^4,31^. An analysis of the NIH-AARP Diet and Health Study found that those living in socioeconomically deprived neighborhoods had a higher risk of CRC-specific mortality, while an additional study of data from the National Center for Health Statistics found that counties experiencing current or PP had increased rates of all cancer and CRC-specific mortality^4,32^. Furthermore, even after controlling for individual-level SES, lifestyle, and medical history, low area-level SES was associated with poorer health and likelihood of an early death, suggesting that residential SES is an independent predictor of overall health outcomes^33^. Interestingly, this association has also been noted in the early-onset (age 20–49) population, where analysis of SEER data showed higher CRC survival for those living in non-poverty, non-rural areas compared to those of age 20–49 in poverty-stricken, rural areas^34^. As the incidence of early-onset CRC is rising and its etiology unclear, understanding the factors that contribute to health outcomes is imperative to combat this public health concern^35,36^. These studies raise the question of why these disparities exist and what can be done to improve patient outcomes.

PP and neighborhood SES are strongly associated with health behaviors and the co-existence of chronic health conditions, underscoring the profound influence of poverty and living conditions on one’s overall health^33,37,38^. Furthermore, CRC disproportionately affects individuals from low SES and impoverished backgrounds in incidence and survival, suggesting potential differences in access to screening and treatment. An evaluation of Missouri Behavioral Risk Factor Surveillance System data found that those living in an area with ≥ 20% poverty rate had lower odds of adherence to CRC screening guidelines, compared to those living in < 20% poverty areas, while a study of individuals living in the Philadelphia metro area found that socioeconomic factors at both the neighborhood and individual levels contributed to low CRC screening completion^37,38^. In the latter study, renting a home was also associated with lower likelihood of screening adherence, which may be correlated with low financial security and income.^38^ Reduced adherence to screening—which may be, at least in part, due to lack of access to cancer screening services—often results in delays in diagnosis and diagnoses at later stages. An analysis of the Ohio Appalachian CRC screening study and Southern Community Cohort Study found that low household income was associated with reduced likelihood of CRC screening^39^. Similarly, individuals living in CRC mortality ‘hotspot regions’, defined as clusters of counties with high rates of CRC-specific mortality, may be less likely to be in compliance with CRC screening guidelines due to area-level deprivation and not solely low income^39,40^. Those residing in intermittent and PP areas were found to present with advanced disease at diagnosis and were less likely to undergo surgery—issues that could be addressed with improved screening access and adherence, as well as access to providers post-diagnosis^41^.

A novel aspect to the current study is the utilization of causal inference to address temporality, specifically that SES census tract precedes, and is a mediator of, PP. Individual-level SES demonstrates concordance with census-tract level SES, and individual SES is dynamic, suggesting that census-tract level SES may be fluid as well, as census-level is the aggregate of the individual^42^. However, those experiencing low SES can experience significant barriers to achieving upward mobility, thereby perpetuating existing inequalities and contributing to the persistence of poverty. This temporal association (SES—> PP) reveals strategic opportunities for targeted interventions, such as improving access to education, employment, healthcare, and food, among other factors, promoting upward mobility, reducing long-term poverty, and, as demonstrated in this study, supporting better health outcomes. By temporally separating chronic persistent poverty from contemporaneous SES and aligning our causal adjustment set accordingly, we avoided the attenuation and inflation seen when these highly correlated constructs are mis-specified. This distinction clarifies that interventions must address both entrenched deprivation and present-day resource deficits.

The current study has several limitations to note. First, as we were restricted to the information provided in SEER, we were not able to derive residential history or comorbidities for the eligible participants, the presence of which may influence treatment options and mortality outcomes. With this in mind, we were also not able to account for changing neighborhood exposure due to residential mobility, screening history, prior colonoscopy, or individual history of polyps, all factors which may contribute to CRC risk. However, a primary strength of our study lies in our analytical method of using causal inference and utilizing PP, as opposed to solely the inclusion of area-level SES, in the presented analyses, as individual and neighborhood SES can generationally change, while PP, as a geographic measurement, incorporates the long-term pattern of poverty.

## Health implications

This study offers valuable insights into the impact of PP on all-cause and CRC-specific mortality, serving as a foundation for further investigation into the root causes of these health inequities.

## Supplementary Information

Supplementary Information 1.

Supplementary Information 2.

Supplementary Information 3.

## Data Availability

All data used in this study are publicly available from the National Institutes of Health and National Cancer Institute. Statistical code will be available upon request to the co-author, Dr. Mohamed Elsaid.

## Declarations

### Reporting guidelines

STROBE guidelines were adhered to during the completion of this study.
