# Supplementary figures and images for: Neighborhood level socioeconomic disparities are associated with reduced colorectal cancer survival
Source: Sci Rep. 2025 Sep 25;15:32795. doi: 10.1038/s41598-025-17659-x (PMC12464318; doi:10.1038/s41598-025-17659-x)

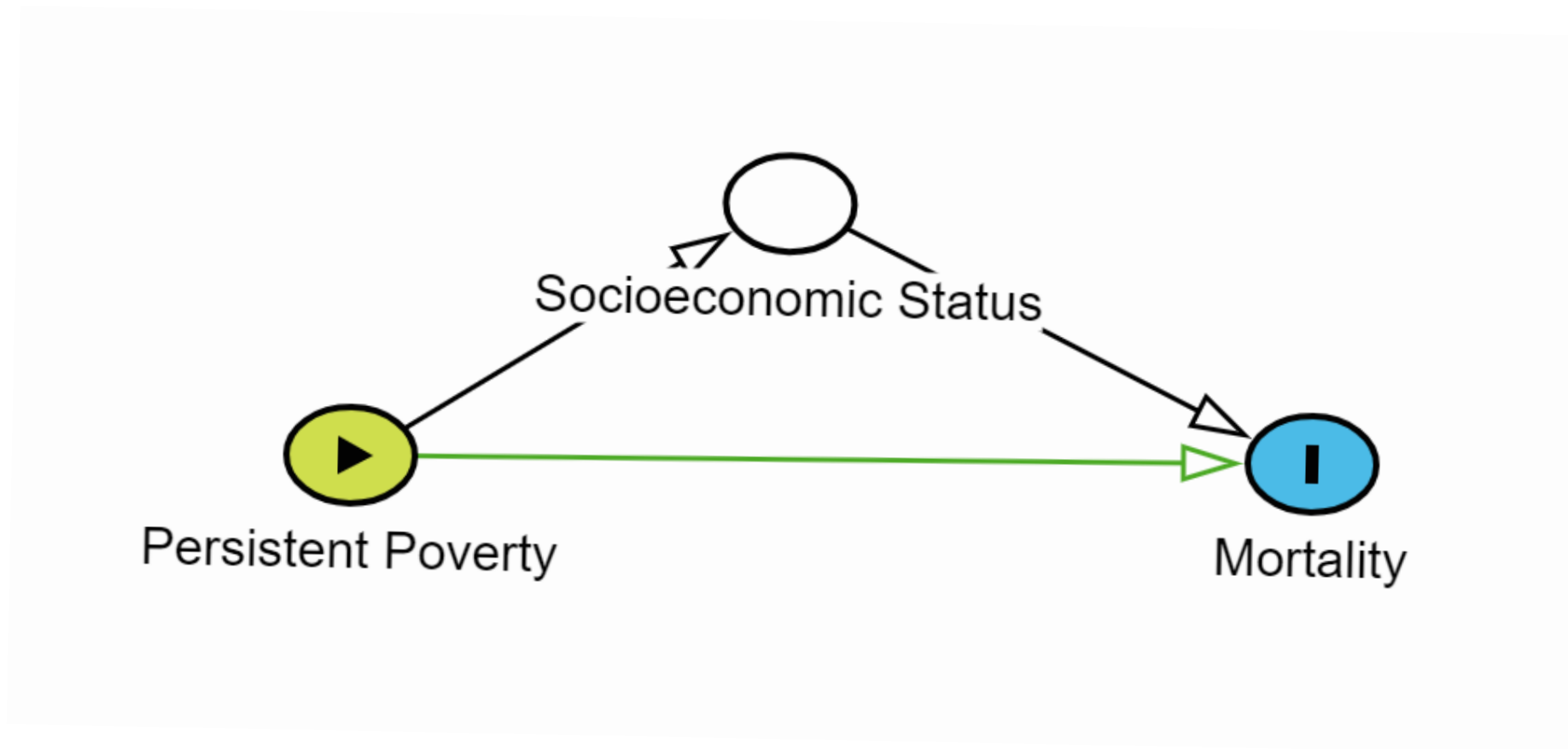

Supplementary Figure S1

**A**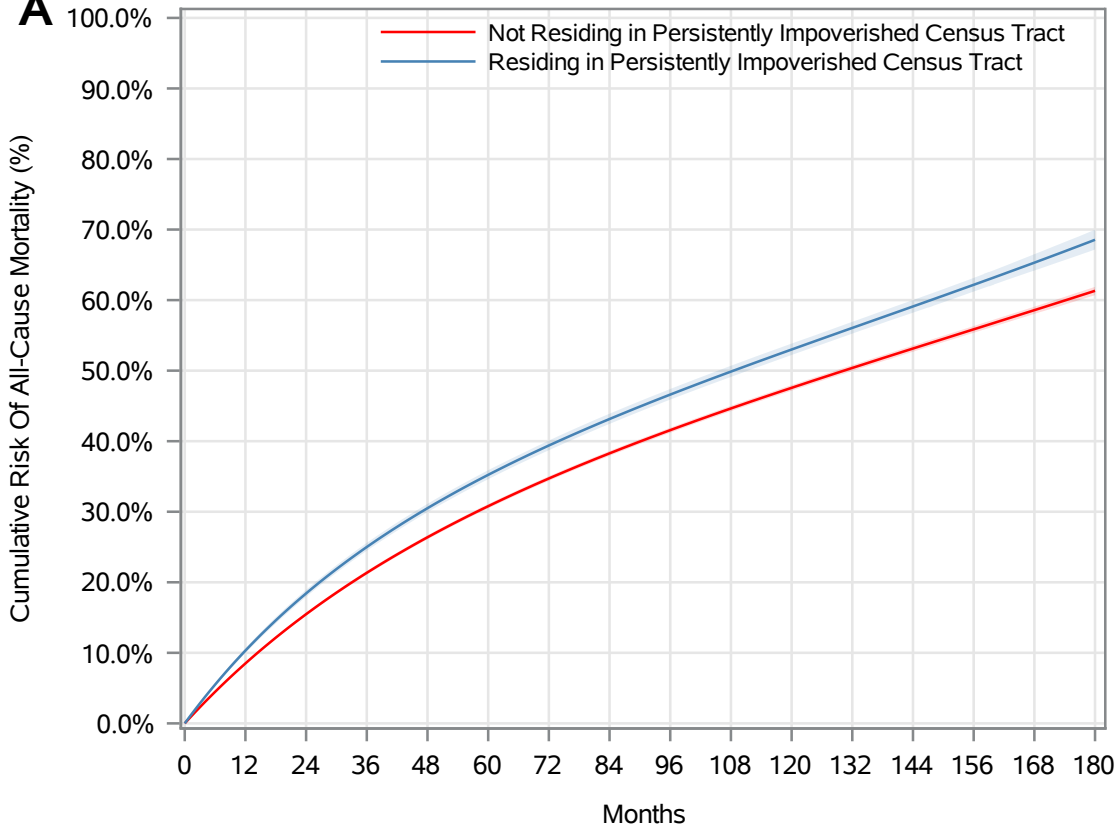**Supplementary Figure S2**

**B**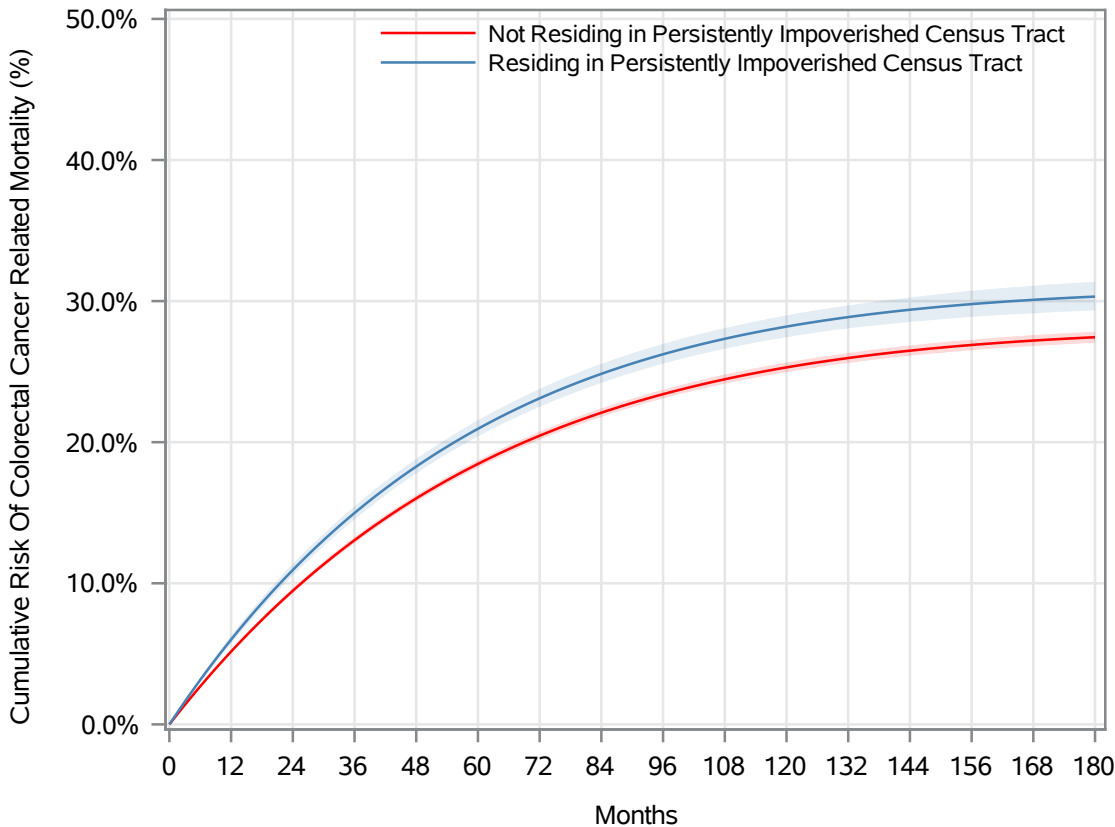

C

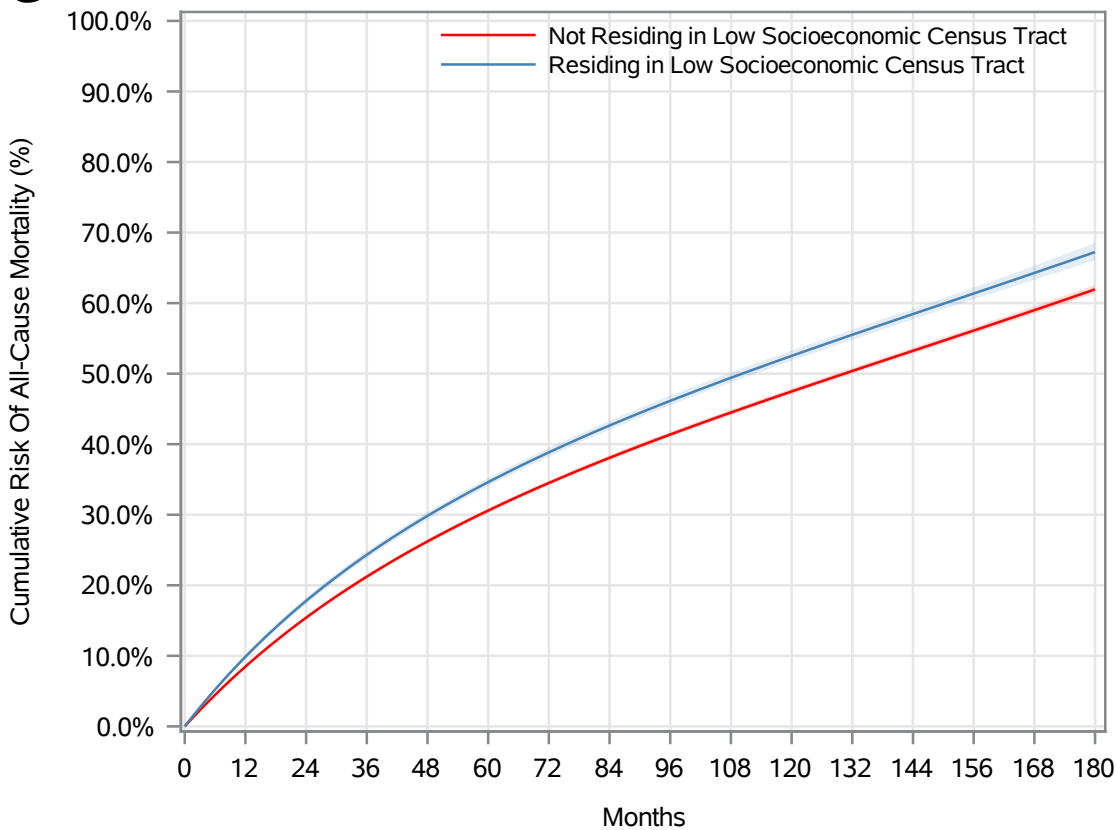

**D**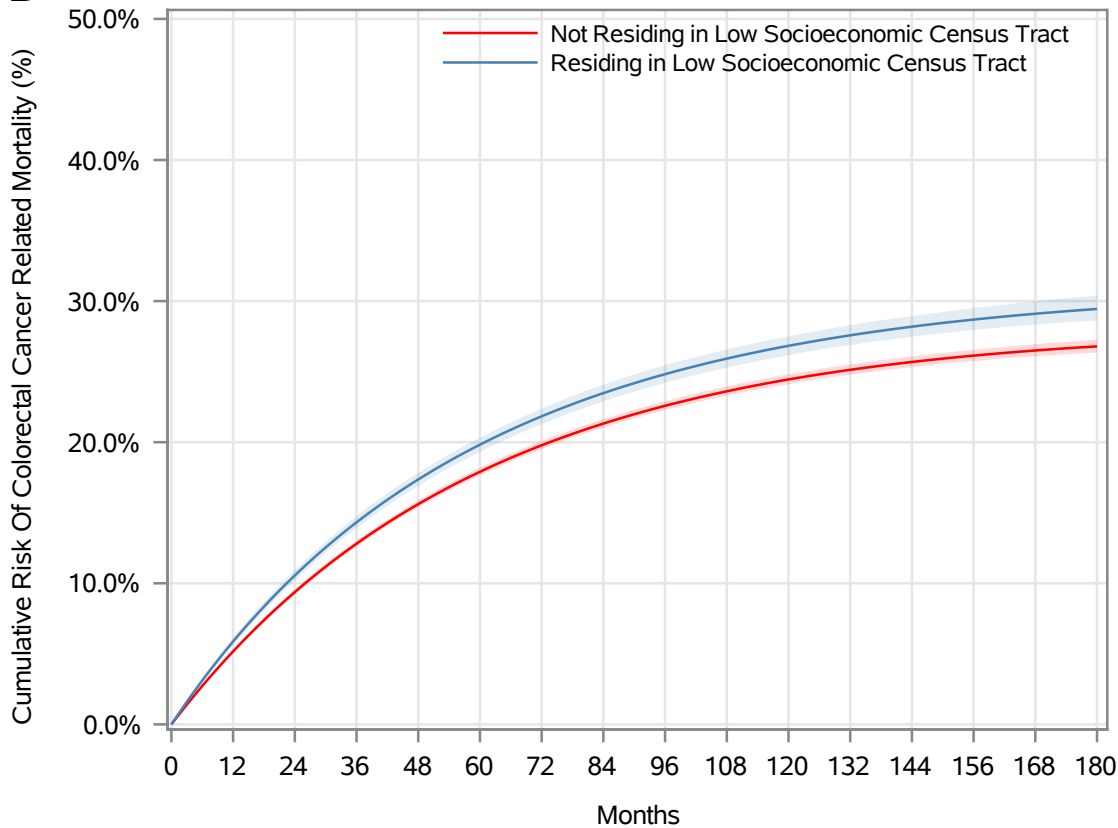

Supplement: Supplementary file 1 — Supplementary Information 1. [file 41598_2025_17659_MOESM1_ESM.pdf]
