# Supplementary material for: Neighborhood level socioeconomic disparities are associated with reduced colorectal cancer survival
Source: Sci Rep. 2025 Sep 25;15:32795. doi: 10.1038/s41598-025-17659-x (PMC12464318; doi:10.1038/s41598-025-17659-x)
Supplement: Supplementary file 2 — Supplementary Information 2. [file 41598_2025_17659_MOESM2_ESM.docx]

**SUPPLEMENTARY MATERIAL**

**Supplementary Table S1.** Sociodemographic, clinical, and treatment characteristics of patients with CRC by residence in low socioeconomic census tract status from the Surveillance, Epidemiology, and End Results (SEER) Program data 2006–2020 (n=253,141).

**Supplementary Table S2**. Associations Between Residing in 1) Persistently Impoverished Census Tract or 2) Low Socioeconomic Census Tract and All-Cause and Colorectal Cancer Specific Mortality in Patients with Colorectal Cancer, The Surveillance, Epidemiology, and End Results (SEER), 2006–2020.

**Supplementary Table S3**. Associations Between Residing in 1) Persistently Impoverished Census Tract or 2) Low Socioeconomic Census Tract and All-Cause and Colorectal Cancer Specific Mortality in Patients with All Stages (Stages I-IV) Colorectal Cancer, The Surveillance, Epidemiology, and End Results (SEER), 2006–2020.

**Supplementary Figure S1.** Directed acyclic graph describing the relationship between persistent poverty, socioeconomic status and mortality.

**Supplementary Figure S2.** Cumulative risk of mortality, assessed by exposure of (1) persistent poverty and (2) socioeconomic status census tract (Yost index). Populations were stratified by residence in persistent poverty (versus not residing in persistent poverty) for (A) all-cause and (B) CRC-specific mortality and residing in low socioeconomic census tracts (versus not residing in low socioeconomic census tracts) for (C) all-cause and (D) CRC-specific mortality.
